# Applications for natural deep eutectic solvents in Chinese herbal medicines

**DOI:** 10.3389/fphar.2022.1104096

**Published:** 2023-01-09

**Authors:** Minghui Li, Cheng Rao, Xiaoqian Ye, Mei Wang, Boyuan Yang, Chengxiao Wang, Liqun Guo, Yin Xiong, Xiuming Cui

**Affiliations:** ^1^ Faculty of Life Science and Technology, Kunming University of Science and Technology, Kunming, China; ^2^ Yunnan Key Laboratory of Panax notoginseng, Kunming University of Science and Technology, Kunming, China; ^3^ Leiden University–European Center for Chinese Medicine and Natural Compounds, Institute of Biology Leiden, Leiden University, Leiden, Netherlands; ^4^ SU Biomedicine BV, Leiden, Netherlands; ^5^ Center for Drug Discovery & Technology Development of Yunnan Traditional Medicine, Kunming, China

**Keywords:** Chinese herbal medicines, NADES, extraction, green solvent, traditional processing (*Paozhi*)

## Abstract

Chinese herbal medicines (CHMs), with a wide range of bioactive components, are considered to be an important source for new drug discovery. However, the process to isolate and obtain those bioactive components to develop new drugs always consumes a large amount of organic solvents with high toxicity and non-biodegradability. Natural deep eutectic solvents (NADES), a new type of green and designable solvents composed of primary plant-based metabolites, have been used as eco-friendly substitutes for traditional organic solvents in various fields. Due to the advantages of easy preparation, low production cost, low toxicity, and eco-friendliness, NADES have been also applied as extraction solvents, media, and drug delivery agents in CHMs in recent years. Besides, the special properties of NADES have been contributed to elucidating the traditional processing (also named *Paozhi* in Chinese) theory of CHMs, especially processing with honey. In this paper, the development process, preparation, classification, and applications for NADES in CHMs have been reviewed. Prospects in the future applications and challenges have been discussed to better understand the possibilities of the new solvents in the drug development and other uses of CHMs.

## 1 Introduction

Chinese herbal medicines (CHMs) have been used to prevent and treat diseases based on the theory of traditional Chinese medicine (TCM) since ancient times. They are mainly derived from different parts of medicinal plants, including roots, stems, leaves, fruits, *etc*. On the one hand, CHMs are an important source of various bioactive components (Rácz et al., 2012). Youyou Tu, a Chinese scientist who discovered artemisinin used to treat malaria, won the Nobel Prize in Physiology or Medicine in 2015. Her discovery was based on TCM knowledge and considered to be a breakthrough in 20th tropical medicine, saving millions of lives in South China, Southeast Asia, Africa, and South America. Therefore, she stated that the discovery of artemisinin against malaria, is a gift from TCM. On the other hand, to isolate and obtain those bioactive components, or to develop new drugs from CHMs, often consumes large amount of organic solvents, most of which are volatile, flammable, and corrosive. Some of them are even highly toxic, carcinogenic, and non-biodegradable, which would be harmful for both the environment and ecosystem (Bushnell et al., 2007).

In the 21st century, a new type of green solvents has been given increased attention. They are different from conventional organic solvents with toxicity, flammability, or pungent odor. Instead, they are composed of components from natural products, which are easy to access, low-cost, non-toxic, and eco-friendly with good biocompatibility and biodegradability. These green solvents were firstly named as “natural deep eutectic solvents (NADES)” in 2011 and considered to be new substitutes for traditional organic solvents and hazardous solvents (Choi et al., 2011). NADES are generated by mixing the hydrogen bond acceptor (HBA) and hydrogen bond donor (HBD) together in a certain proportion. Both HBA and HBD in the binary system of NADES originate from biosynthetically primordial metabolites, including sugars, amino acids, choline, and some organic acids (Liu et al., 2018). The melting point of the mixture is lower than those of the individual components, which is the most typical feature of NADES. Hydrogen bondings and van der walls interactions are the main driving forces of this phenomenon (Espino et al., 2016).

Although the research of NADES is comparatively in its infancy, they have gradually become more popular and been used in various fields, such as electrochemistry, nanotechnology, catalysis, and biomedical research (He et al., 2019). Since the properties of NADES depend largely on the components and could be adjustable, they have been recently applied to extract and isolate various natural bioactive compounds from CHMs, including phenols, flavonoids, terpenoids, and alkaloids (Duan et al., 2016; Zeng et al., 2018). Also, they have been used as adjuncts to improve the stability, oral bioavailability, and skin permeability of some CHMs, thereby enhancing the therapeutic effect of these drugs (Xuan et al., 2021). Interestingly, the properties of NADES have even provided evidences of molecular interactions for elucidating traditional *Paozhi* theory of processing CHMs with honey. To understand the current status and foresee new possibilities of combing the novel solvents and traditional medicines, this study provides an overview of applications for NADES in CHMs. We hope to provide a clue for developing and utilizing CHMs from a new direction, and developing new drugs from natural resources in a more efficient, eco-friendly, and sustainable way.

## 2 “Green” solvents and development of NADES

**FIGURE 1:**
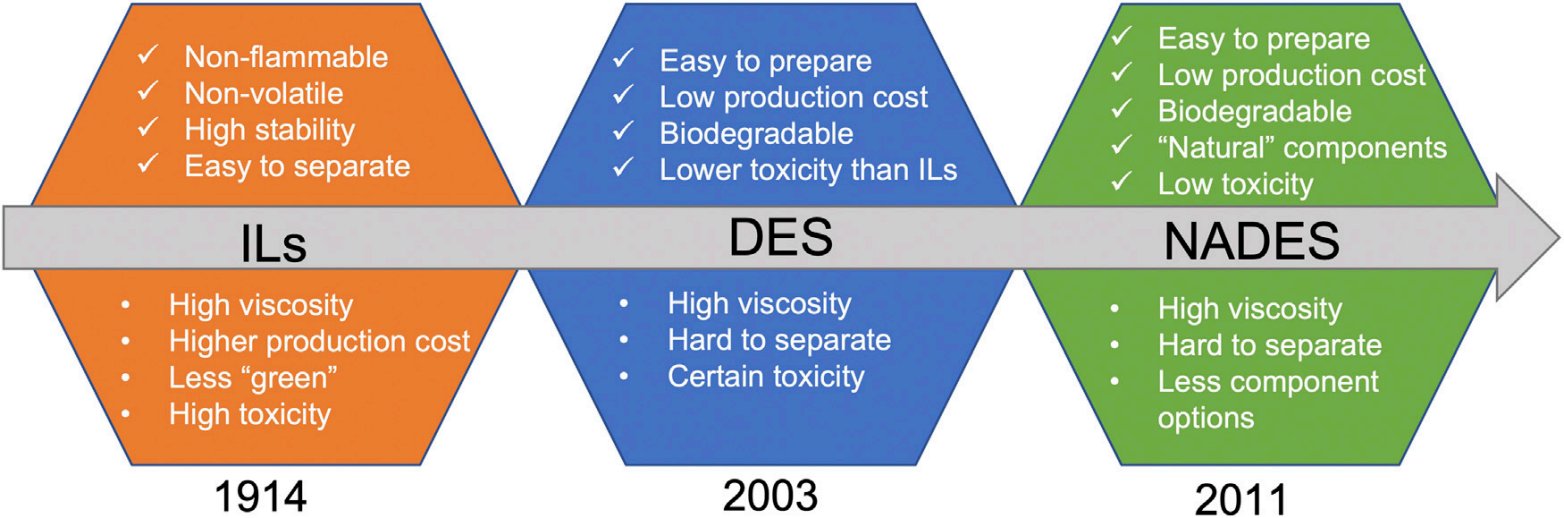
Timeline of reported developments and properties of ILs, DES, and NADES. DES, deep eutectic solvents; ILs, ironic liquids; NADES, natural deep eutectic solvents.

The idea of “green” solvents expresses the goal to minimize the environmental impact resulting from the use of solvents in chemical production (Capello et al., 2007). Ionic liquids (ILs), known as the initial green solvents, were reported by Walden in 1914 for the first time (Walden, 1914) (Figure 1). ILs are liquid molten salts at temperatures below 100°C or even at room temperature, which are formed from systems composed generally of organic cations and organic or inorganic anions. It is well known that ILs are non-flammable, non-volatile, and stable in air and water (Brennecke and Maginn, 2001). The preparation of ILs mainly consists of two steps: i) protonation of amines to cations, and ii) using Lewis acids to treat halide salts or by anion decomposition reactions. However, the byproducts and wastes generated from ILs make it less “green”. They often cause the persistent pollutants in the wastewater which are difficult to be removed because of the high stability of ILs in water. Meanwhile, some studies have shown that the compounds of imidazole structure as constituents of some ILs showed significant toxicity (Romero et al., 2008).

In order to overcome the drawbacks of ILs, deep eutectic solvents (DES) were introduced as the alternatives and were first proposed in 2003 (Abbott et al., 2003). DES are generally synthesized by gently heating the mixture while simply mixing the ingredients that can form clear and stable liquid solutions at room temperature, in a shorter amount of time (Florindo, et al., 2014). Carboxy, hydroxyl, and carbonyl are the most abundant functional groups of DES components. For example, choline chloride (ChCl), acetylcholine chloride, and various amino acids can be used as HBAs (Smith et al., 2014) and other compounds such as urea, lactic acid (LA), and citric acid (CA) (Abbott et al., 2004) are often used as the HBDs. Different from ILs that the ionic interactions are the main interaction forces, the components of DES interact with each other through the hydrogen bondings (Kelley et al., 2013). The reasons that ILs can be replaced by DES mainly include the easily available and cheap ingredients, and the easy and time-saving preparation process. However, subsequent studies have shown that the potential toxicity and cytotoxicity may be imparted by various DES. For instance, Hayyan et al. (2013) found that the cytotoxicity of some phosphonium-based DES was higher than their individual components and that their overall toxicity was varied depending on the structures of the latter. Therefore, the physicochemical properties, biological effects, and toxicological profiles of DES could be potentially adjusted by using different types of components (Juneidi et al., 2016).

In 2011, Choi et al. proposed the concept of NADES, which solely consist of natural components, i.e., primary metabolites (e.g., sugars, amino acids, organic acids, polyols, and tertiary amines) (Choi et al., 2011). The nuclear magnetic resonance metabolomics showed that in all organisms certain ingredients for NADES are present as major compounds, often keeping a constant molar ratio to some others. This brought the hypothesis that “Everywhere in living systems NADES occur and form a third liquid phase of intermediate polarity”. Apart from water and lipids, NADES aid certain cells in biosynthesis and storage of bioproducts, cryoprotection, and drought resistance (Choi et al., 2011). Compared to conventional organic solvents, NADES are considered as “green” solvents in terms of the biodegradability and sustainability, and the toxicity is generally lower than that of traditional DES (Fuad et al., 2021). Currently, NADES have been proposed as potential excipients in pharmaceutical preparations and drug delivery systems, particularly because of their solubilizing properties, varying viscosities, and built-in bioactivities (Grønlien et al., 2020).

## 3 Preparation of NADES

**FIGURE 2:**
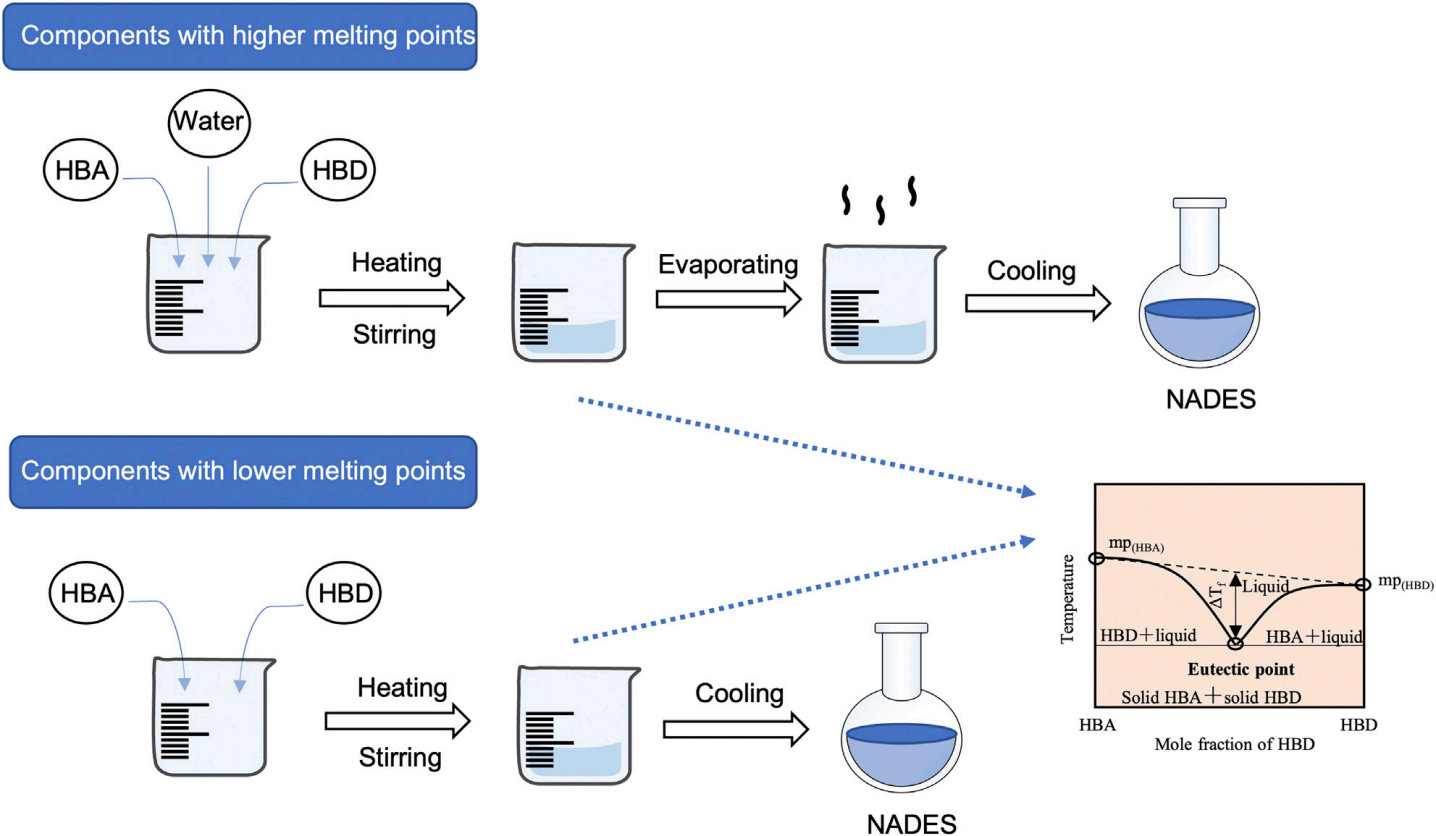
Preparation of NADES with components with higher/lower melting points. HBA, hydrogen bond acceptor; HBD, hydrogen bond donor; NADES, natural deep eutectic solvents.

NADES are prepared by mixing two or more common natural products in a specific molar ratio that form a liquid (Figure 2). In general, the components of NADES are commercially available. For components with higher melting points, a small amount of water is usually added into the NADES mixture by a certain molar ratio. Then the mixture is heated while stirred at a certain temperature until a clear and homogeneous liquid is obtained. In the last stage, the residual water can be removed through evaporation in vacuum or the combined use of other techniques such as freeze drying (Oomen et al., 2020).

For NADES components with lower melting points, the water is not necessary and the NADES can be prepared by directly mixing and constantly stirring two or three components in certain molar ratios under proper temperatures (e.g. ChCl and LA at the molar ratio of 1:1 at 60°C). Sometimes the heating should be performed with magnetic agitation to obtain a homogeneous transparent liquid. After cooling the mixture to room temperature, the transparency and homogeneity of the mixture could indicate that the designed ratio is feasible. In addition, all NADES prepared need to be kept in a desiccator before use (Xie et al., 2019).

Due to the difference in the properties of various components to prepare NADES, the modification to the preparation process is usually inevitable. Parameters such as the heating time, heating temperature, heating stability, and water ratio should be adjusted according to different properties of the components.

## 4 Classification of NADES

**FIGURE 3:**
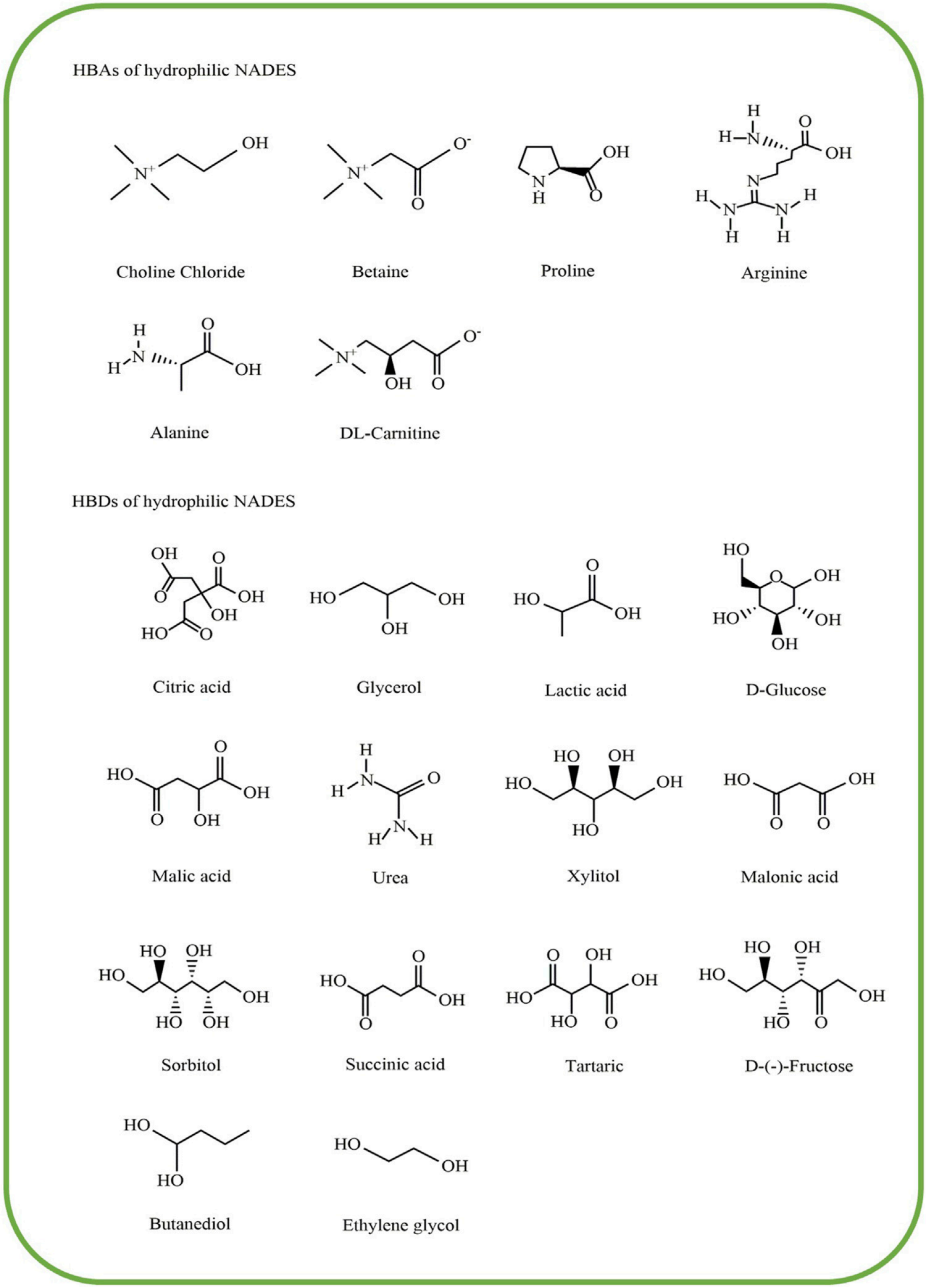
Common components of hydrophilic NADES. NADES, natural deep eutectic solvents.

The hydrophilicity and hydrophobicity of NADES are essential indicators for evaluating their properties and applications. NADES without a clear prefix are identified as hydrophilic NADES (Figure 3) due to their prominent position in various studies and the richer source from the nature. For example, the primary metabolites of sugars [e.g., glucose (Glu), fructose (Fru), sucrose (Suc), and trehalose] in nature, amino acids [e.g., proline (Pro)], and organic acids [e.g., malic acid (MA) and CA], etc., are all candidates for the hydrophilic and designable eutectic compositions. The density of hydrophilic NADES is reported to be related to the molecular weight of HBAs and HBDs. The higher the molecular weight of the components is, the higher the density is (Fuad et al., 2021). For example, the hydrophilic NADES containing sugars and organic acids have relatively higher density, owing to their higher molecular weight. In comparison, the density of hydrophilic NADES containing alcohols is usually lower. Also, hydrophilic NADES often have higher viscosity. According to different types of HBDs, the viscosity of ChCl-based NADES and betaine (Bet)-based NADES was increased as follows: alcohols < organic acids and sugar. But NADES containing LA often have a relatively low viscosity (Freitas et al., 2022).

**FIGURE 4:**
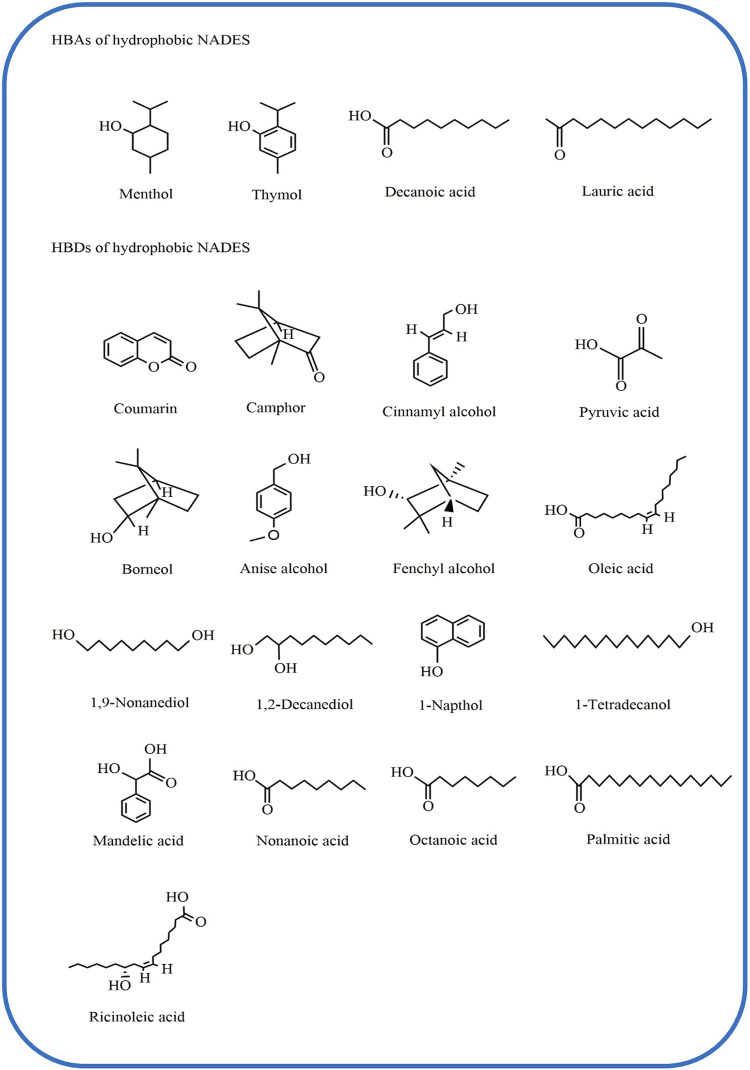
Common components of hydrophobic NADES. NADES, natural deep eutectic solvents.

In contrast, the studies on hydrophobic NADES (Figure 4) are much less than that on hydrophilic NADES. van Osch et al. (2019) proposed four criteria for judging and evaluating the hydrophobic NADES, including i) the viscosity less is than 100 mPa s; ii) the difference between the density of NADES and the density of water is more than 50 kg m^−3^; iii) when NADES are mixed with water, a low transfer rate of NADES to the water phase should be presented; and iv) there is little or no change in pH value. The substances commonly used in the synthesis of hydrophobic NADES involve menthol (Men), thymol, coumarin, camphor, etc. (Martins et al., 2018), which have lower solubility in the water phase. The hydrophilicity and hydrophobicity of NADES depend on the carbon chain length of HBA and HBD. The longer the carbon chain contained in the structure is, the stronger the most hydrophobic effect expresses (Florindo et al., 2019). For example, L-Men, belonging to hydrophobic natural component with long alkyl chains, was often used as HBA to synthesize hydrophobic NADES (Ribeiro et al., 2015). Some bioactive compounds in CHMs, e.g. carthamin and rutin, showed poor solubility in water (Dai et al., 2014; Zhao, et al., 2015). Therefore, the hydrophobic NADES were used as the medium for dissolving and extracting them.

## 5 Current applications for NADES in CHMs

### 5.1 Extraction of active components in CHMs

Water, as the most widely used and easily accessed solvent in the extracting process of CHMs, is a good candidate to extract active components with strong polarity (Lu and Jiang, 2013). However, there are multiple bioactive components in CHMs with strong lipophilicity which exhibit very low solubility in water (Wang et al., 2018). Other traditional organic solvents such as methanol, ethanol, and ethyl acetate are also common solvents for extracting bioactive substances (Chemat et al., 2019). But many of them are volatile, flammable, explosive, and even toxic (Basant et al., 2016). In recent years, NADES have been designed to extract various bioactive compounds from CHMs due to their better biocompatibility, higher solubility, and subsequently higher extraction yields sometimes (Table 1). By investigating results in Table 1, we can find that the type of NADES components, molar ratio, water content, and several extraction conditions are major factors that would affect the extraction yield.

**TABLE 1:** Extraction of active compounds from CHMs by NADES.

No.	Plant name of CHM and medicinal part	Target compounds	Bioactivities	NADES composition and molar ratio	Water content (v/v)	Extraction technique	Liquid-solid ratio	Temperature	Time	Evaluation result	References
1	*Pueraria lobata* (Willd.) Ohwi, root	Puerarin (PUE), 3′-methoxypuerarin, and puerarin-6″-O-xyloside	Hepatoprotection, and improving cardiovascular functions	Pro-MA, 1:1	40%	Ultrasound-assistance extraction (UAE)	40 mL/g	40°C	30 min	The extraction yields of PUE, 3′-methoxypuerarin and puerarin-6″-O-xyloside were 98.7 mg/g, 16.3 mg/g and 9.9 mg/g, respectively, which were 2.2-, 2.9- and 3.4-fold higher than that of water	Huang et al. (2021)
2	*Scutellaria baicalensis* Georgi, root	Baicalein, scutellarein, wogonin, aoroxylin, baicalin, scutellarin, wogonoside, and oroxyloside	Decreasing blood pressure, antitoxin, and antifever	Alanine–CA, 1:1; Pro–CA, 1:1	50% 40%	UAE	20 mL/g	40°C	30 min	The extraction yields of flavonoids with NADES were 2–6 times than that of aqueous methanol. The extraction yields of glycosides with NADES was 1.5–1.8 times higher than with aqueous methanol	Oomen et al. (2020); Guo et al. (2007); Kowalczyk et al. (2006); Li et al. (2011)
3	*Carthamus tinctorius* L., flower	Hydroxysafflor yellow A, cartormin, and carthamin	Antioxidation, promoting blood circulation, and removing blood stasis	Suc−ChCl; Pro−MA; LA− Glu	25% 25% ---	Heating and stirring	30 mL/g	40°C	60 min	The extraction yields of hydroxysafflor yellow A and cartormin with Pro-MA were 8% and 14% higher than 40% ethanol, respectively; the extraction yield of carthamin with LA-Glu was 8% and 14% higher than 40% ethanol	Dai et al. (2013); Lu et al. (2019); Liao et al. (2018)
4	*Curcuma Longa* L., root	Curcumin	Antioxidation, anti-inflammation, and anticarcinma	Fru-ChCl-water, 2:5:5; Suc-ChCl-water, 1:4:4; Fru-LA-water, 1:5:5; Suc-LA-water, 1:5:7; LA-ChCl-water, 1:1:2		Microwave-assistance extraction (MAE)	72.5–82.5 mL/g	64.7°C–71.8°C	15.4–21.6 min	The contents of curcumin in all NADES extracts were higher than 80% methanol (Fru-ChCl-water, 2:5:5)	Doldolova et al. (2021); Aggarwal et al. (2007); Allegra et al. (2017)
5	*Glycyrrhiza glabra* L., root and rhizome	Glycyrrhizic acid (GA)	Anti-allergy, antivirus, and hepatoprotection	ChCl-LA, 1:1	30%	UAE	30 mL/g	---	15 min	In comparison with the NADES stirring method, the NADES-UAE technique reduced the extraction time of GA by 50% and solvent consumption by 25%	Lanjekar and Rathod. (2021); Tian et al. (2008)
6	*Artemisia annua* L., stem and leaf	Artemisinin	Antimalaria	Carnitine-isosorbide, 1:2	---	UAE	20 mL/g	48°C	32.62 min	The extraction yield of artemisinin was 1.1954 mg/g	Pan et al. (2021); Pandey and Pandey-Rai. (2016)
7	*Abrus cantoniensis* Hance, stem	Total flavonoids	Antivirus, lowering blood pressure, and hepatoprotection	ChCl-ethylene glycol, 1:2	30%	UAE	15 mL/g	80°C	40 min	The extraction rates of total flavonoids and total saponins were increased by 33.3% and 96.4%, respectively, compared with traditional solvents	Chen et al. (2019)
		Total saponins		ChCl-LA, 1:4	25%		56 mL/g	80°C	64 min		
8	*Rhodiola crenulata* (Hook. f. et Thoms.) H. Ohba, rhizome	Rosavin, salidroside, rosin, cinnamyl alcohol, and tyrosol	Anti-tumor and anti-inflammation	LA-Fru-water, 5:1:11		UAE	40 mL/g	22°C	154 min	The extraction yields of salidroside, tyrosol, rosavin, rosin, cinnamyl alcohol, and total markers were 11.90 ± .02, .36 ± .02, 12.23 ± .21, 1.41 ± .01, .20 ± .01, and 26.10 ± .27 mg/g, respectively	Shikov et al. (2020)
9	*Fraxinus stylosa* Lingelsh., bark	Coumarins (aesculetin, aesculin, fraxetin, and fraxin)	Antivirus, anti-inflammation, and anticancer	Bet-glycerin (Bet-Gly), 1:3	20%	UAE	67 mL/g	---	30 min	The extraction rate with NADES was significantly increased than with conventional solvents	Wang et al. (2020a); Srikrishna et al. (2018)
10	Jinqi Jiangtang Preparations	Neochlorogenic acid, chlorogenic acid, groenlandicine, isochlorogenici, coptisine, and berberine	Anti-diabetics	ChCl-laevulinic acid, 1:2	50%	UAE	125 mL/g	---	60 min	ChCl-laevulinic acid (1:2) was more effective than the methanol–water solution for the simultaneous extraction of multi-compounds with a wide range of polarity	Yang et al. (2019a); Yang et al. (2019b): Cao et al. (2010)
11	*Trollius chinensis* Bunge, flowers	Orientin, vitexin, and 2″-O-galactopyra-nosylorientin	Antimicrobe and antivirus	ChCl-zinc bromide, 1:1	48%	UAE	42 mL/g	---	28 min	Under the optimum conditions, the extraction yields of total flavone-C-glycosides were 14.97 mg/g	Duan et al. (2018); Cai et al. (2006)
12	*Salvia miltiorrhiza* Bge., root and rhizome	Salvianolic acid B (SAB), tanshinone IIA, and cryptotanshinone	Anti-blood coagulation, neuroprotection, and anti-cancer	Pro-LA, 1:1	25%	UAE	100 mL/g	50°C	30 min	The yields of SAB, tanshinone IIA, and cryptotanshinone were 42.05 mg/g (53% higher than methanol), 1.485 mg/g, and .839 mg/g, respectively	He et al. (2019); Chen et al. (2016); Fan et al. (2018); Zhang et al. (2019)
13	*Sophora japonica* L., flower	Rutin	Anti-inflammation and antivirus	ChCl-Gly, 1:1	20%	UAE				The extraction yield of rutin with ChCl-Gly was the highest, reaching 291.57 mg/g	Zang et al. (2020)
14	*Coptis chinensis* Franch., rhizome	Columbamine, jatrorrhizine, epiberberine, coptisine, palmatine, and berberine	Lowering cholesterol and lowering blood pressure	Bet-tartaric acid-water, 1:1:1		UAE	35 mL/g		29.5 min	The extraction yield was 128.43 ± .03 mg/g	Li et al. (2020); Kong et al. (2004)
15	*Dioscoreae nipponica* Makino, rhizome	Protodioscin, protogracillin, pseudoprotodioscin, and pseudoprotogracilli -n	Relieving cough, eliminating rheumatic aches, and improving blood circulation	ChCl-malonic acid, 1:1	40%	UAE	32 mL/g	---	20 min	The extraction yield of the total four steroidal saponins was 66.82 mg/g	Yang et al. (2021); Ou-Yang et al. (2018)
16	*Penganum harmala* L., seed	Harmine	Neuroprotection, enhancing cognition, and anti-inflammation	Men-anise alcohol, 1:1	---	Centrifugation	50 : 1 (volume of water phase: volume of extractant phase)	25°C	5 min	The extraction rate of Men- anise alcohol (1:1) for harmine was higher than the traditional organic solvents and ILs	Fan et al. (2020)
17	*Ligusticum chuanxiong* Hort, root	Ferulic acid	Promoting blood circulation and removing blood stasis	ChCl-1,2-propanediol (ChCl-PG), 1:2	30%	MAE	30 mL/g	68°C	20 min	The extraction yield of ferulic acid with NADES-MAE (2.32 mg/g) was higher than that with 73% ethanol-heating (1.52 mg/g) and 40% ethanol-MAE (.77 mg/g)	Xie et al. (2019); Lu et al. (2019); Liao et al. (2018)
18	*Rheum palmatum* L., root and rhizome	Total anthraquinones (aloe-emodin, rhein, emodin, chrysophanol. and physcion)	Anti-inflammation, antivirus, and anti-obesity	LA-Glu, 5:1	10%	UAE	26 mL/g	82°C	90 min	The extraction yield of the total anthraquinones obtained with LA-Glu (5:1) was 1.8 times higher than chloroform	Wu et al. (2018); Kim et al. (2010); Chang et al. (2014); Li et al. (2016)

#### 5.1.1 NADES components

According to Table 1, the ChCl-containing NADES were the most frequently-used solvents to extract bioactive compounds from CHMs, indicating that ChCl, especially as HBA, played an important role in this application. Additionally, more than half of the NADES were prepared with organic acids (e.g. MA, CA, and LA), which were more favorable for the extraction of alkaloids. The extraction yields using NADES with organic acids as HBDs were relatively higher than those containing sugars or alcohols (Li et al., 2020). Also, the organic acid-containing NADES exhibited an excellent extraction capacity when used to extract steroidal saponins (Yang et al., 2021) and phenolics (Dai et al., 2013), which are two representative components with various activities in CHMs. For extracting phenolic metabolites in the CHM of *Carthamus tinctorius* flower, Pro-MA and LA-Glu were found to be more efficient than 40% ethanol in the extraction process (Dai et al., 2014), which might be attributed to the formation of strong hydrogen bonds between molecules of NADES and phenolic compounds.

#### 5.1.2 Molar ratio of NADES components

The physicochemical properties of NADES, e.g. viscosity, surface tension, and polarity, would change along with the variation in the molar ratios of NADES components, which often affect the extraction efficiency (Tian et al., 2008; Guo et al., 2019). In most cases in Table 1, NADES had the positive properties when the molar ratio of HBA and HBD was 1:1 or 1:2 when extracting bioactive components from CHMs. For example, ChCl-PG of 9 M ratios, *i.e.* 1:0.2,1:0.25, 1:0.33, 1:0.5, 1:1, 1:2, 1:3, 1:4, and 1:5 were prepared to extract ferulic acid from *Ligusticum chuanxiong* Hort. The results showed that ChCl-PG at the ratio of 1:2 had the highest extraction rate (Xie et al., 2019). The thermodynamic analysis showed that the hydrogen bond in NADES was the main force in the extraction process (Fan et al., 2020). And NADES with components at a certain ratio often came with a suitable viscosity, which thereby brought an increased number of hydrogen bonds and stronger force during the extraction. However, the optimized molar ratios for different NADES components were varied. Different ratios of the targeted NADES should be initially prepared to determine the optimal one.

#### 5.1.3 Water content of NADES

Generally, NADES have higher viscosity than conventional solvents. And the water content of NADES is a key factor affecting the viscosity and the followed extraction yield (Craveiro et al., 2016; Wang et al., 2020b). NADES with higher viscosity would hinder the dissolution of bioactive components. In order to improve the extraction efficiency of NADES, a certain amount of water could be added to reduce the viscosity. For example, the extraction yield of phenolic compounds was increased along with the increased water content of Suc-ChCl from 10% to 25% (Dai et al., 2013). However, the water content of NADES and the extraction yield of target compounds were not in a linear relationship in most cases. The optimal extraction capacity of NADES could be only reached at a specific water content. Xie et al. investigated the extraction capacity of ChCl-PG (1:2) with a water content from 0% to 60%. The results showed that the extraction yield of ferulic acid was initially increased and then decreased, reaching a maximum extraction yield when the water content was 30% (Xie et al., 2019). The reason might be that the excessive water in NADES would increase the polarity of the solvent, whereas some compounds were difficult to be dissolved in solvents of high polarity (Wu et al., 2018). In addition, some research revealed that when the water content of NADES reached or exceeded 50%, it would negatively affect the stability of NADES. In such system, hydrogen bonds would be broken and the NADES components were completely dissociated and hydrated. Consequently, the physical and chemical properties of NADES would be altered. And the extraction yields of the target compounds were then decreased (Cui et al., 2018).

#### 5.1.4 Extraction technique

UAE was the most used extraction technique to assist NADES in extracting bioactive components from plants. This might be due to its advantages of energy saving, high efficiency, safety, and environmental friendliness compared with other extraction methods (Cunha and Fernandes, 2018). According to results in Table 1, UAE was applied in more than 75% of the studies to extract components from CHMs with NADES. The higher extraction rate was considered to be related to the damaged structure of CHMs by the strong mechanical effect of ultrasound, making it more favorable for solubilizing large quantities of compounds into NADES (Li et al., 2020). Besides UAE, MAE was another commonly used extraction method, by which the microwaves with extremely high frequencies could penetrate the solvent and reach the cells inside the solid raw materials (Chan et al., 2016). The advantage of this method was that high levels of the target compounds could be obtained in a much shorter amount of time. The water in cells absorbed the energy from microwaves, which increased the temperature and pressure inside cells, resulting in cell disruption and dissolution of active components. However, MAE was frequently performed under higher temperature, which could affect the stability of active components (Zuo et al., 2021).

#### 5.1.5 Extraction temperature

The extraction temperature often has a significant influence on the extraction yield of components from CHMs. Apart from adding a certain percentage of water, increasing the temperature was also a way to reduce the viscosity of NADES (Liu et al., 2018). Along with the increase of temperature, the energy of the molecules would be increased, which enabled them to dissolve and diffuse faster in the liquid media (Francisco et al., 2013). However, for the active molecules with poor thermal stability, they could be decomposed or even inactivated at higher temperatures. For example, the important peroxide group in the structure of artemisinin was decomposed under the influence of high temperature, which led to a lower extraction yield (Pan et al., 2021). Therefore, the determination of a suitable temperature is necessary in the extraction process.

#### 5.1.6 Extraction time

In most cases, the yield of target compounds could be increased with a longer extraction time, and then maintained constant. For example, after the extraction time over 30 min, the extraction yield of artemisinin was hardly increased (Pan et al., 2021). The extraction yield of coumarin also reached the maximum at 30 min, and then was almost constant at 40 min and 50 min. This might be due to that the target bioactive components were completely extracted, or the compounds in the solvent were saturated. In some cases, there would be a declined phase, which mainly depended on the properties of the target bioactive components. When the extraction time or the exposure duration of active compounds to air was too long, they could be oxidized or decomposed, thereby resulting in a reduction of the extraction yield. The study from Xie et al. (2019) confirmed that a long extraction time was not suitable for the extraction of ferulic acid. Also, the energy consumption was another factor to consider to determine the extraction time. The extraction time in most of the studies summarized in Table 1 was less than 60 min.

#### 5.1.7 Liquid-solid ratio

Studies have shown that a higher liquid-solid ratio could generally produce a positive effect on the extraction yield of compounds. With the increase of liquid-solid ratio, the solid matrix would have more contact with the solvent, which was beneficial for the dissolution of the target compounds. However, such positive effect could only be maintained at a certain range of the liquid-solid ratio. For example, the ratio of Bet-tartaric acid-water (1:1:1) to *Coptis chinensis* rhizome powder at 35 mL/g obtained the highest extraction yield of alkaloids. And then the adding of NADES failed to increase the extraction yield significantly (Li et al., 2020). Similar result could be found in another study, that the extraction yield of GA was increased with the increase of liquid-solid ratio from 10 to 30 mL/g. However, when the liquid-solid ratio reached 40 mL/g, the extraction yield was decreased, which might be due to a cavitation effect between the excessive solvent and the solid powder (Lanjekar and Rathod, 2021). Moreover, the higher ratio of solvent is not cost effective in the industrial scale.

### 5.2 Improving the bioavailability of CHMs

#### 5.2.1 Improving the oral bioavailability of active components from CHMs

Oral administration is the most common way of administration of CHMs. However, some active components from CHMs with low bioavailability can be poorly absorbed by human body when taken orally. For example, paeoniflorin showed strong anti-depression activity. But its oral bioavailability was determined as 2.32% (Yu et al., 2019). According to the Biopharmaceutics Classification System II substances, the bioavailability may be enhanced by increasing the solubility and dissolution rate of the drug in the gastrointestinal fluids (Khadka et al., 2014). NADES are green solvents which have been applied in increasing the solubility and oral bioavailability of some components through the hydrogen-bond interaction with drugs.

Chen et al. (2017) evaluated the influence of ChCl-GL (1:2) on the pharmacokinetics of SAB, one of the active components from *Salvia miltiorrhiza* Bge. used to relieve the pain and calm the mind in TCM. They found that compared with water, ChCl-GL could promote the absorption of SAB, showing a higher peak concentration and shorter peak time than dissolving SAB in water. Another example is about berberine, a quaternary benzylisoquinoline alkaloid in many Chinese medicinal plants such as *Berberis spp*. and *Coptis spp*. Despite its demonstrated therapeutic efficacies in anti-inflammatory, anti-microbial, and cholesterol-lowering treatments, the orally administered berberine can be poorly absorbed and rapidly undergone extensive metabolism (Spinozzi et al., 2014). Sut et al. (2017) investigated the pharmacokinetic profiles of berberine solubilized in three considered NADES after orally administered to mice. The results revealed a 2–20 folds increase of berberine level in blood concentration when treating with NADES-berberine compared with the treatment of water-berberine. The similar trend was found in another pioneering work by Farggian et al. (Faggian et al., 2016), that the NADES of Pro-glutamic acid significantly enhanced the maximum concentration and the area under curve of rutin in Balb/c mice. It revealed an increase in the relative bioavailability of rutin in the NADES formulation of approximately 100% when compared with its behavior in the corresponding aqueous solution. Also, 25 different NADES were prepared for the extraction of PUE and its two natural derivatives from the dried root of *Pueraria lobata* (Willd.) Ohwi (Huang et al., 2021). According to the results, the optimized NADES increased the extraction yields of PUE and its derivatives by 2.2–3.4 folds compared with water. Furthermore, the relative oral bioavailability of PUE in NADES was 323%, which was even higher than the nanocrystal self-stabilized Pickering emulsion and self-microemulsifying drug-delivery systems of PUE (Qiao et al., 2018; Zhang et al., 2018).

#### 5.2.2 Promoting the skin-permeation of CHMs

Among various administration routes of drugs, the transdermal drug delivery system (TDDS) is favored due to its advantages of improved bioavailability, more uniform plasma levels, reduced side effects and first-pass drug degradation effects because of the maintenance of plasma levels up to the end of the dosing interval compared with a decline in plasma levels by conventional oral dosage forms (Patel et al., 2012). It has been reported that NADES can significantly enhance the percutaneous absorption of small molecules as well as macromolecular drugs, such as proteins, small interfering RNAs, and polysaccharides in TDDS systems (Berton et al., 2017; Qu et al., 2019).

Inspired by these applications, Xiao et al. (2022) developed a novel hydrogel TDDS incorporating an amino acid-based NADES for the CHM product of “Sanwujiaowan”, which is original prepared as pills for oral administration to treat rheumatoid arthritis and rheumatoid myositis. Sanwujiaowan is composed of the extracts of five CHM ingredients, some of which contain components with certain degree of toxicity. By preparing the NADES-extract complex, it was found that there were excellent dissolution and skin permeability of the components in the extracts. Moreover, the consequent hydrogel with the NADES-extract complex exerted an enhanced therapeutic effect that significantly reduced the inflammatory response with systemic toxicity of the extracts. Wang et al. (2017) prepared a microemulsion based on the NADES of paeonol and Men, which are active components from the root bark of *Paeonia suffruticosa* Andr. and the herb of *Mentha haplocalycis* Briq, respectively. The results showed that the microemulsion-based gel formulations facilitated the drug permeation compared with the simple mixture gel of paeonol and Men without forming a NADES system.

### 5.3 Improving the stability of active components in CHMs

NADES could significantly enhance the stability of some bioactive components in CHMs, e.g. phenolic compounds (Dai et al., 2013), without weaking their activities. *Carthamus tinctorius* L. is a CHM widely used in the clinic to treat cardiovascular diseases, which owns efficacies of invigorating the circulation of blood, dissipating blood stasis, and relieving pains (Kim and Paik, 1997). Carthamin is one of the major active components in *Carthamus tinctorius* L, which has strong antioxidant activity (Jeliński et al., 2019). However, carthamin is unstable in aqueous solutions (Sut et al., 2017) and also sensitive to the change of temperature (Ji et al., 2016). It was found that the sugar-based NADES with lower water content could significantly improve the stability of carthamin under various conditions such as high temperature, light exposure, and long storage time, compared with other solvents of water and 40% ethanol (Dai et al., 2014). This increased stability was attributed to the high viscosity and low mobility of NADES. The free carthamin molecules were encapsulated tightly, thereby restricting the movement of the solute molecules and enhancing their stability, which was consistent with previous conclusion that NADES containing sugar generally possessed higher viscosity. Similarly, Wikene et al. (2015) investigated the effect of NADES on the stability of curcumin, an active component in the CHM of *Curcuma longa* L, which is sensitive to light and temperature under normal conditions. They synthesized several NADES and found that the hydrolytic stability of curcumin in NADES was comparable to or up to 2–10 times higher than in cyclodextrins and up to >1300 times higher than in the buffer solution at pH 8.

### 5.4 Elucidation of possible molecular bases for traditional processing theory of CHMs

Processing of CHMs, also named *Paozhi*, is a treatment to improve the medical applications of CHMs in TCM. Its functions include altering the flavor, moderating the medicinal properties, and eliminating the side effects or toxicities of the crude materials. The importance of processing materials has already been mentioned in the Huang Di Nei Jing (The Yellow Emperor’s Internal Classic, 475–221 B.C.) and Shen Nong Ben Cao Jing (Divine Husbandman’s Classic of the Materia Medica, *c*. 220 A.D.) (Chang et al., 2011). In the current TCM practice, all the materials are strictly required to be properly processed before using for therapeutic application. The techniques of *Paozhi* include cutting, crushing, steaming, calcining, stir-frying, with or without adjuvants (Dai et al., 2021). Honey is one of the most common adjuvants in the stir-frying of *Paozhi*, which could keep its liquidity even with a low water content and was hypothesized to be a sugar-based NADES. The stir-frying with honey often begins with the dilution of boiled water. Then the honey solution is added to the cleaned and cut plant materials, mixed thoroughly, kept for a while and then stir-fried with gentle heat until the materials get a specific color (Chinese Pharmacopoeia Committee, 2020). This process has the following effects according to TCM theory: 1) moistening lungs for relieving cough (e.g. Stemono Radix); 2) enhancing the tonifying effects on spleen and nourishing qi (e.g. Astragali Radix); 3) moderating the properties of raw materials (e.g. Ephedra Herba); and 4) correcting the flavor and eliminating the side effects of drugs (e.g. Aristolochia Fructus) (Jin, 1988).

To prove the hypothesis that honey behaves like a NADES in the process of *Paozhi*, Dai et al. (2021) processed Astragali Radix with or without honey in different ways. They found that the level of isoflavonoids and saponins were far more increased by honey and NADES treated samples than in the decoction of raw materials. It indicated that the artificial honey using a sugar-based NADES, had the same effect as real honey, which supported the hypothesis that honey, similar as NADES, could improve the solubility of medium polar plant secondary metabolites present in the plant materials. Consequently, the bioeffects of honey and NADES on estrogen receptor, androgen receptor, and antioxidant-related factors were significantly improved than those of the raw material decoction, which was consistent with the traditional function of honey in *Paozhi*. Based on a series of studies, it was suggested that the mechanisms of honey in the stir-frying process, behaving like NADES, might promote the conversion of aglucones to glucosides, inhibit the hydrolysis of the glucosides, inhibit acetylation of isoflavonoids and astragalosides, increase deacetylation of acetyl-containing isoflavonoids and astragalosides, inhibit the hydrolysis of the malonylated and/or acetylated glucosides, and improve the extraction of the isoflavonoids glucosides if compared to the aglycones (Ma et al., 2009; Zheng et al., 2014).

## 6 Prospects of future applications for NADES in CHMs and challenges

### 6.1 Prospects of future applications

#### 6.1.1 Development of active pharmaceutical ingredient (API)-NADES formulations

**FIGURE 5:**
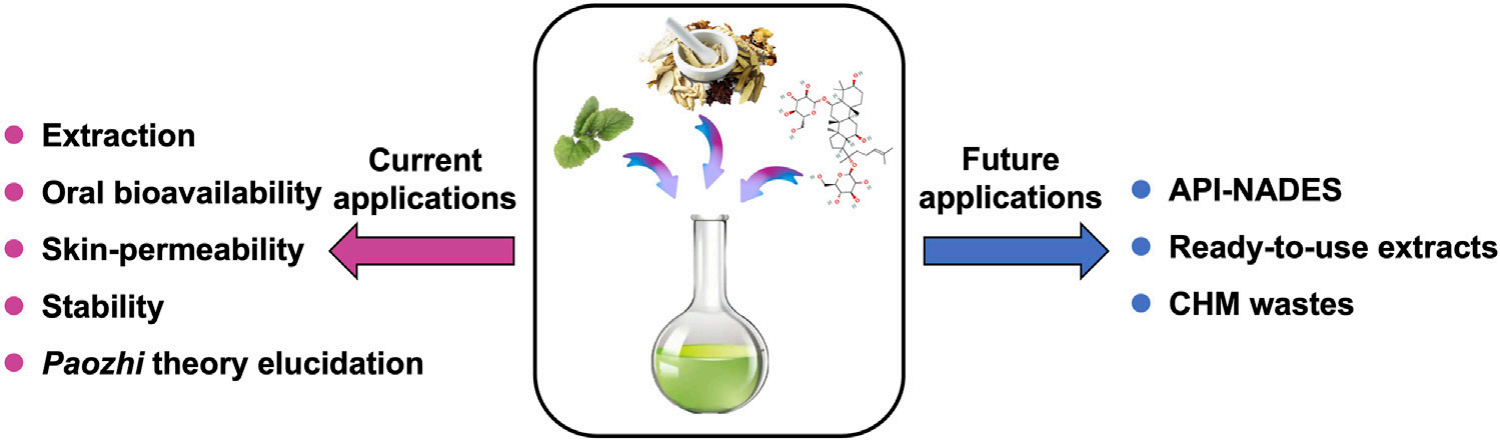
Current and future applications for NADES in CHMs. CHMs, Chinese herbal medicines; NADES, natural deep eutectic solvents.

CHMs are great source of multiple APIs which have potential to be developed as drugs. Unlike the previous reviewed applications (Figure 5) that NADES were mostly used as solvents for APIs in CHMs, it is promising that APIs from CHMs could be directly used as NADES phase-forming constituents by acting as HBDs and/or HBAs, to exhibit higher therapeutic effects or improved properties through their own eutectic systems, *i.e*. API-NADES. The formulation of API-NADES could usually improve the solubility and permeability of the APIs in comparison to their isolated solid forms, as the liquid state of the APIs confers them an enhanced bioavailability and thus, better efficacy (Roda et al., 2020). In the study from Wang et al. (2017), the permeation performance of paeonol was significantly increased in its eutectic mixture with methanol. The reasons might be that: i) the NADES mixture may cause the leaching of the lipids in the skin and thereby formation of pores, and/or ii) it may depress the melting point of drug to below skin temperature thereby increase the drug solubility (Park et al., 2012). Due to the property as a permeation enhancer, Men has been widely used in API-NADES formulations with chemical drugs such as ibuprofen and aspirin (Yong et al., 2003; Aroso et al., 2016), which increased folds of permeation capability of those drugs compared to the pure form of them.

In some other cases, the formulation could also improve the stability of APIs and even promote a controlled release to achieve the therapeutic effect. In the work from Mano et al. (2017), the API-NADES composed by ChCl-mandelic acid (1:2) and encapsulated in gelatin showed a fast-dissolving release profile in phosphate buffered saline without cytotoxicity. The formulation also maintained the anti-bacterial effect of mandelic acid on both Gram-positive and negative bacteria. Inspired by these applications, it is rather promising to develop API-NADES formulations based on various components from CHMs to improve their targeted properties, such as solubility, bioavailability, stability, or fast drug delivery capacity to the target site.

#### 6.1.2 Development of ready-to-use NADES extract of CHMs

NADES represent a new green strategy to overcome the toxicity and environmental disadvantages of conventional organic solvents, which have been demonstrated as efficient as conventional organic solvents for the extraction of multiple components from fruits, seeds, flowers, leaves, and other parts of plants (da Silva et al., 2021). In the meantime, the solvents in many of those NADES extracts did not need to be removed, and no toxicity was observed from the NADES *per se*. Thus, such NADES mixtures have the potential to be used as ready-to-use extracts for the development of functional foods, nutraceuticals, or cosmetic products.

In the study from Komaikul et al. (2021), they extracted stilbenoids from *Morus alba* callus by using a selected NADES of chloride-GL mixture. The NADES extract of *Morus alba* callus showed comparable anti-melanogenesis activity compared with methanol extract and no irritation effect on reconstructed human *epidermis*. After 6 months of storage, the stilbenoids in the NADES remained stable at the storage temperature of 4°C. It suggested NADES could be advantageous for the preparation of skin-lightening cosmetics without removing the solvent. Besides being an eco-friendly and non-irritable solvent for the extraction of active phytochemicals, some NADES showed potential to be used as a ready-to-use vehicle for increasing oral absorption of bioactive compounds. For example, da Silva et al. (2021) investigated if a ready-to-use extract obtained using a NADES affects the pharmacokinetic profile of blueberry phenolic compounds compared to organic solvent-extracted compounds. The non-compartmental pharmacokinetic analysis revealed that the NADES extract increased the bioavailability of anthocyanins by 140% compared to the organic solvent extract. And the stability of phenolic compounds in NADES was increased during *in vitro* digestion by delaying gastric chyme neutralization.

Those strategies could be referred for the development of products based on CHMs. For CHMs, a large number of them have been investigated for the development of cosmetic products with anti-oxidative, anti-wrinkle, skin lightening and other types of skin-care functions; or pharmaceuticals to treat or prevent various diseases. Therefore, the combination of CHMs or their components with NADES, especially the preparation of related ready-to-use extracts, could be a direction for the further development of products used in the cosmeceutical or pharmaceutical, or even nutraceutical industries.

#### 6.1.3 Utilization of CHMs waste with NADES

Based on TCM theory, only certain parts of medicinal herbs are harvested and processed as CHMs in the clinic. For example, the main root of *Panax ginseng* Meyer is traditionally and widely accepted as the medicinal part of this CHM, whereas other parts, e.g. fibrous roots, leaves, flower buds, and fruits, were hardly used. However, according to the chemical analysis, the content of ginsenosides, the major active components of *Panax ginseng* Meyer, was higher in the leaf and fibrous roots (Shi et al., 2007). Similar results were found in *Panax notoginseng* (Burk.) F. H. Chen, the protopanaxadiol type saponins were rich in the leaves and flowers, whereas rare in the underground parts (Wan et al., 2006). Those findings suggested that the sustainable development and rational utilization of CHMs waste or non-medicinal parts would be necessary and might increase the economic value of related industries.

To recover active components from agro-industrial wastes or convert those wastes into a wide range of bio-based products based on the biorefinery concept, NADES have been employed in a number of studies and shown an advantageous potential to utilize the biowastes. In the study of Bertolo et al. (2021), NADES were used as alternative solvents to recover the phenolic compounds from the pomegranate peel waste. Under optimal extraction conditions, the phenolic recovery rate with ChCl and LA was increased by 54.6%, when compared with the non-optimized conditions, and by 84.2%, when compared with the ethanolic extraction. In another biorefinery research, Kumar et al. (2018) conducted a comprehensive evaluation of an integrated process for cellulosic ethanol production from a NADES based pretreated rice straw. They obtained the maximum ethanol yield with a theoretical conversion efficiency of 79.9%. These studies demonstrated the potential of NADES as an effective environmentally friendly alternative for obtaining valuable compounds from agro-industrial waste, and the applicability to be used in the economically viable integrated biorefinery process. Considering the commonality in the material base and processing method of many CHMs waste and agro-industrial one, those applications with NADES could be learned for the better utilization of CHMs waste in the future.

### 6.2 Challenges of applying NADES in CHMs

Even though NADES offer several benefits as compared with traditional solvents, e.g. lower toxicity, higher extractability of diverse compounds with varied polarity, and capacity to improve the bioactivity and stability, there are still challenges and limits when used in CHMs due to their inherent properties.

Due to the high viscosity and low vapor pressure of NADES, the recycling and reusing of NADES after the extraction process is the major challenge to develop a cost-effective extraction process, which might impede its application at industrial scale or in continuous-flow reactions. In most cases, the resins or molecular sieves were used to recover active components from the NADES extract, which also involved the use of large number of alternative solvents. When those alternative solvents were organic ones, this process seems to, eventually, counterbalance the benefits of using NADES. Sometimes the addition of antisolvents (ethanol or water) is probably the most valuable method since it does not require a specific equipment or additional investment and allows the processing in a sustainable way. After extracting the solutes by precipitation or by forming an ethanol- or water-insoluble layer, the initial NADES could be recovered by removing ethanol or water through evaporation. Yet, it cannot be used for ethanol- or water-soluble NADES. Therefore, further research in the final recycling or purification step should be a future focus to implement an efficient, eco-compatible, and sustainable extraction of APIs from CHMs with NADES as the solvents. Another possible solution could be the development of ready-to-use extracts as what has been proposed in Section 6.1.2, which does not need to remove the solvents.

Moreover, even though the components of NADES are generally considered to be safe and non-toxic, there is no comprehensive toxicity evaluation *in vivo* of different NADES yet. According to an acute toxicity assessment by using mice (Mbous et al., 2017), the investigated NADES exhibited a certain degree of hepatotoxic effect. This was attributed to the viscosity of these solvents, which might have failed to circulate properly in mice, and then blocked and halted blood flow. Another risk might be induced by the increased permeability of NADES. Based on the results from the same study above, the NADES treated cells had an increased porosity compared with the control cells without NADES treatment, suggesting an increased capacity of NADES to perforate cellular membranes. On the one hand, this property might increase the therapeutic effects of possible NADES-CHMs extracts as discussed before. On the other hand, it could be also possible to increase the toxicity of those formulations due to the increased permeability of NADES. Therefore, besides the accurate selection of HBA and HBD for optimizing the extraction of targeted APIs in a CHM, the toxicity evaluation of the NADES and the corresponding NADES-CHM extract should be also an integral part in further studies.

## 7 Conclusion

CHMs have been used to prevent and treat diseases for thousands of years, of which the medical claims are supported by clinical evidence of a long history. With the development of extraction and purification technology, more and more bioactive compounds have been identified and obtained from CHMs, making them an important resource for the discovery of APIs. In the meantime, CHMs have their own unique features. For example, they are administered under the guidance of TCM theory and often given in formulars. NADES, as novel green solvents, are mostly used to extract multiple phytochemicals and food components, showing advantages over the organic solvents, such as enhanced bioactivity of bioactive compounds and their higher stability in NADES under various storage conditions. Their special properties offer the possibility for the development of CHMs as well as related products, including extracting APIs in a more effective way, increasing the stability, bioavailability, and skin-permeability of components or extracts from CHMs, and even shedding light on the elucidation of traditional *Paozhi* theory. The applications of NADES in cosmeceutical, pharmaceutical, and nutraceutical areas also inspired those in CHMs, such as the preparation of API-NADES formulations, development of ready-to-use CHM-NADES extracts, and utilization of non-medicinal parts from CHM plants. Apart from that, the challenges coexist with the opportunities, which reminds us to pay more attention on research of recycling or recovering the solvents, and the necessary toxicity assessment in the future.

## Abbreviations

API, active pharmaceutical ingredient; Bet, betaine; CA, citric acid; ChCl, choline chloride; CHMs, Chinese herbal medicines; DES, deep eutectic solvents; Fru, fructose; GA, glycyrrhizic acid; GL, glycerol; Glu, glucose; Gly, glycerin; HBA, hydrogen bond acceptor; HBD, hydrogen bond donor; ILs, ionic liquids; LA, lactic acid; MA, malic acid; MAE, microwave-assistance extraction; Men, menthol; NADES, natural deep eutectic solvents; PG, propanediol; Pro, proline; PUE, puerarin; SAB, salvianoli cacid B; Suc, sucrose; TCM, traditional Chinese medicine; TDDS, transdermal drug delivery system; UAE, ultrasound-assistance extraction.

## References

[B1] Abbott A. P. Boothby D. Capper G. Davies D. L. Rasheed R. K. (2004). Deep eutectic solvents formed between choline chloride and carboxylic acids: Versatile alternatives to ionic liquids. J. Am. Chem. Soc. 126, 9142–9147. 10.1021/ja048266j 15264850

[B2] Abbott A. P. Capper G. Davies D. L. Rasheed R. K. Tambyrajah V. (2003). Novel solvent properties of choline chloride/urea mixturesElectronic supplementary information (ESI) available: Spectroscopic data. See http://www.rsc.org/suppdata/cc/b2/b210714g/. Chem. Comm., 70–71. 10.1039/b210714g 12610970

[B3] Aggarwal B. B. Bhatt I. D. Ichikawa H. (2007). Curcumin–biological and medicinal properties. Turmeric: The Genus Curcuma. Boca Raton, FLLondon: CRC Press, 297–368.

[B4] Allegra A. Innao V. Russo S. Gerace D. Alonci A. Musolino C. (2017). Anticancer activity of curcumin and its analogues: Preclinical and clinical studies. Cancer Invest. 35, 1–22. 10.1080/07357907.2016.1247166 27996308

[B5] Aroso I. M. Silva J. C. Mano F. Ferreira A. S. D. Dionísio M. Sá-Nogueira I. (2016). Dissolution enhancement of active pharmaceutical ingredients by therapeutic deep eutectic systems. Eur. J. Pharm. Biopharm. 98, 57–66. 10.1016/j.ejpb.2015.11.002 26586342

[B6] Basant N. Gupta S. Singh K. P. (2016). Predicting the acute neurotoxicity of diverse organic solvents using probabilistic neural networks based QSTR modeling approaches. Neurotoxicology 53, 45–52. 10.1016/j.neuro.2015.12.013 26721664

[B7] Bertolo M. R. V. Martins V. C. A. Plepis A. M. G. Bogusz S. (2021). Utilization of pomegranate peel waste: Natural deep eutectic solvents as a green strategy to recover valuable phenolic compounds. J. Clean. Prod. 327, 129471. 10.1016/j.jclepro.2021.129471

[B8] Berton P. Di Bona K. R. Yancey D. Rizvi S. A. A. Gray M. Gurau G. (2017). Transdermal bioavailability in rats of lidocaine in the forms of ionic liquids, salts, and deep eutectic. ACS Med. Chem. Lett. 8, 498–503. 10.1021/acsmedchemlett.6b00504 28523100 PMC5430390

[B9] Brennecke J. F. Maginn E. J. (2001). Ionic Liquids: Innovative fluids for chemical processing. AIChE J. 47, 2384–2389. 10.1002/aic.690471102

[B10] Bushnell P. J. Boyes W. K. Shafer T. J. Bale A. S. Benignus V. A. (2007). Approaches to extrapolating animal toxicity data on organic solvents to public health. Neurotoxicology 28, 221–226. 10.1016/j.neuro.2006.03.013 16684563

[B11] Cai S. Q. Wang R. Yang X. Shang M. Ma C. Shoyama Y. (2006). Antiviral flavonoid-type C-glycosides from the flowers of Trollius chinensis. Chem. Biodivers. 3, 343–348. 10.1002/cbdv.200690037 17193271

[B12] Cao H. Ren M. Guo L. Shang H. Zhang J. Song Y. (2010). JinQi-jiangtang tablet, a Chinese patent medicine, for pre-diabetes: A randomized controlled trial. Trials 11, 27. 10.1186/1745-6215-11-27 20214831 PMC2842259

[B13] Capello C. Fischer U. Hungerbühler K. (2007). What is a green solvent? A comprehensive framework for the environmental assessment of solvents. Green Chem. 9, 927–934. 10.1039/B617536H

[B14] Chan C. H. Yeoh H. K. Yusoff R. Ngoh G. C. (2016). A first-principles model for plant cell rupture in microwave-assisted extraction of bioactive compounds. J. Food Eng. 188, 98–107. 10.1016/j.jfoodeng.2016.05.017

[B15] Chang S. J. Huang S. H. Lin Y. J. Tsou Y. Y. Lin C. W. (2014). Antiviral activity of Rheum palmatum methanol extract and chrysophanol against Japanese encephalitis virus. Arch. Pharm. Res. 37, 1117–1123. 10.1007/s12272-013-0325-x 24395532 PMC7091366

[B16] Chang W. T. Choi Y. H. Van der Heijden R. Lee M. S. Lin M. K. Kong H. (2011). Traditional processing strongly affects metabolite composition by hydrolysis in *Rehmannia glutinosa* roots. Chem. Pharm. Bull. 59, 546–552. 10.1248/cpb.59.546 21532190

[B17] Chemat F. Vian M. A. Ravi H. K. Khadhraoui B. Hilali S. Perino S. (2019). Review of alternative solvents for green extraction of food and natural products: Panorama, principles, applications and prospects. Molecules 24, 3007. 10.3390/molecules24163007 31430982 PMC6721174

[B18] Chen J. Wang Q. Liu M. Zhang L. (2017). The effect of deep eutectic solvent on the pharmacokinetics of salvianolic acid B in rats and its acute toxicity test. J. Chromatogr. B 1063, 60–66. 10.1016/j.jchromb.2017.08.016 28846866

[B19] Chen R. Li D. Ruan G. Liu S. Sun K. Huang S. (2019). Green and efficient extraction of flavoniods and saponins from Abrus cantoniensis Hance by deep eutectic solvents. Nat. Prod. Res. Dev. 31, 1632–1640. 10.16333/j.1001-6880.2019.9.022

[B20] Chen Y. Zhang N. Ma J. Zhu Y. Wang M. Wang X. (2016). A Platelet/CMC coupled with offline UPLC-QTOF-MS/MS for screening antiplatelet activity components from aqueous extract of Danshen. J. Pharm. Biomed. Anal. 117, 178–183. 10.1016/j.jpba.2015.06.009 26355772

[B21] Chinese Pharmacopoeia Committee (2020). Pharmacopoeia of the people’s Republic of China Part I. Beijing: Chinese Medical Science and Technology Press.

[B22] Choi Y. H. van Spronsen J. Dai Y. Verberne M. Hollmann F. Arends I. W. C. E. (2011). Are natural deep eutectic solvents the missing link in understanding cellular metabolism and physiology? Plant Physiol. 156, 1701–1705. 10.1104/pp.111.178426 21677097 PMC3149944

[B23] Craveiro R. Aroso I. Flammia V. Carvalho T. Viciosa M. T. Dionísio M. (2016). Properties and thermal behavior of natural deep eutectic solvents. J. Mol. Liq. 215, 534–540. 10.1016/j.molliq.2016.01.038

[B24] Cui Q. Liu J. Wang L. Kang Y. Meng Y. Jiao J. (2018). Sustainable deep eutectic solvents preparation and their efficiency in extraction and enrichment of main bioactive flavonoids from sea buckthorn leaves. J. Clean. Prod. 184, 826–835. 10.1016/j.jclepro.2018.02.295

[B25] Cunha S. C. Fernandes J. O. (2018). Extraction techniques with deep eutectic solvents. Trends Anal. Chem. 105, 225–239. 10.1016/j.trac.2018.05.001

[B26] da Silva D. T. Smaniotto F. A. Costa I. F. Baranzelli J. Muller A. Somacal S. (2021). Natural deep eutectic solvent (NADES): A strategy to improve the bioavailability of blueberry phenolic compounds in a ready-to-use extract. Food Chem. 364, 130370. 10.1016/j.foodchem.2021.130370 34182361

[B27] Dai Y. Choi Y. H. Verpoorte R. (2021). Honey in traditional Chinese medicine: A guide to future applications of NADES to medicines. Adv. Botanical Res. 97, 361–384. 10.1016/bs.abr.2020.09.011

[B28] Dai Y. Verpoorte R. Choi Y. H. (2014). Natural deep eutectic solvents providing enhanced stability of natural colorants from safflower (*Carthamus tinctorius*). Food Chem. 159, 116–121. 10.1016/j.foodchem.2014.02.155 24767033

[B29] Dai Y. Witkamp G. J. Verpoorte R. Choi Y. H. (2013). Natural deep eutectic solvents as a new extraction media for phenolic metabolites in *Carthamus tinctorius* L. Anal. Chem. 85, 6272–6278. 10.1021/ac400432p 23710664

[B30] Doldolova K. Bener M. Lalikoğlu M. Aşçı Y. S. Arat R. Apak R. (2021). Optimization and modeling of microwave-assisted extraction of curcumin and antioxidant compounds from turmeric by using natural deep eutectic solvents. Food Chem. 353, 129337. 10.1016/j.foodchem.2021.129337 33752120

[B112] Duan L. Dou L. L. Guo L. Li P. Liu E. H. (2016). Comprehensive evaluation of deep eutectic solvents in extraction of bioactive natural products. ACS Sustain. Chem. Eng. 4, 2405–2411. 10.1021/acssuschemeng.6b00091

[B31] Duan L. Zhang W. H. Zhang Z. H. Liu E. H. Guo L. (2018). Evaluation of natural deep eutectic solvents for the extraction of bioactive flavone C-glycosides from Flos Trollii. Microchem. J. 145, 180–186. 10.1016/j.microc.2018.10.031

[B32] Espino M. Fernández M. D. L. A. Gomez F. J. V. Silva M. F. (2016). Natural designer solvents for greening analytical chemistry. Trends Anal. Chem. 76, 126–136. 10.1016/j.trac.2015.11.006

[B33] Faggian M. Sut S. Perissutti B. Baldan V. Grabnar I. Dall’Acqua S. (2016). Natural deep eutectic solvents (NADES) as a tool for bioavailability improvement: Pharmacokinetics of rutin dissolved in proline/glycine after oral administration in rats: Possible application in nutraceuticals. Molecules 21, 1531. 10.3390/molecules21111531 27854256 PMC6272970

[B34] Fan Y. Luo Q. Wei J. Lin R. Lin L. Li Y. (2018). Mechanism of salvianolic acid B neuroprotection against ischemia/reperfusion induced cerebral injury. Brain Res. 1679, 125–133. 10.1016/j.brainres.2017.11.027 29180227

[B35] Fan Y. Wu H. Cai D. Yang T. Yang L. (2020). Effective extraction of harmine by menthol/anise alcohol-based natural deep eutectic solvents. Sep. Purif. Technol. 250, 117211. 10.1016/j.seppur.2020.117211

[B36] Florindo C. Branco L. C. Marrucho I. M. (2019). Quest for green-solvent design: From hydrophilic to hydrophobic (deep) eutectic solvents. ChemSusChem 12, 1549–1559. 10.1002/cssc.201900147 30811105

[B37] Florindo C. Oliveira F. S. Rebelo L. P. N. Fernandes A. M. Marrucho I. M. (2014). Insights into the synthesis and properties of deep eutectic solvents based on cholinium chloride and carboxylic acids. ACS Sustain. Chem. Eng. 2, 2416–2425. 10.1021/sc500439w

[B38] Francisco M. Van Den Bruinhorst A. Kroon M. C. (2013). Low-transition-temperature mixtures (LTTMs): A new generation of designer solvents. Angew. Chem. Int. Ed. 52, 3074–3085. 10.1002/anie.201207548 23401138

[B39] Freitas D. S. Rocha D. Castro T. G. Noro J. Castro V. I. B. Teixeira M. A. (2022). Green extraction of cork bioactive compounds using natural deep eutectic mixtures. ACS Sustain. Chem. Eng. 10, 7974–7989. 10.1021/acssuschemeng.2c01422

[B40] Fuad F. M. Nadzir M. M. Harun A. (2021). Hydrophilic natural deep eutectic solvent: A review on physicochemical properties and extractability of bioactive compounds. J. Mol. Liq. 339, 116923. 10.1016/j.molliq.2021.116923

[B41] Grønlien K. G. Pedersen M. E. Tønnesen H. H. (2020). A natural deep eutectic solvent (NADES) as potential excipient in collagen-based products. Int. J. Biol. Macromol. 156, 394–402. 10.1016/j.ijbiomac.2020.04.026 32289414

[B42] Guo N. Kou P. Jiang Y. W. Wang L. T. Niu L. J. Liu Z. M. (2019). Natural deep eutectic solvents couple with integrative extraction technique as an effective approach for mulberry anthocyanin extraction. Food Chem. 296, 78–85. 10.1016/j.foodchem.2019.05.196 31202309

[B43] Guo Q. Zhao L. You Q. Yang Y. Gu H. Song G. (2007). Anti-hepatitis B virus activity of wogonin *in vitro* and *in vivo* . Antivir. Res. 74, 16–24. 10.1016/j.antiviral.2007.01.002 17280723

[B44] Hayyan M. Hashim M. A. Al-Saadi M. A. Hayyan A. AlNashef I. M. Mirghani M. E. (2013). Assessment of cytotoxicity and toxicity for phosphonium-based deep eutectic solvents. Chemosphere 93, 455–459. 10.1016/j.chemosphere.2013.05.013 23820537

[B111] He X. Yang J. Huang Y. Zhang Y. Wan H. Li C. (2019). Green and efficient ultrasonic-assisted extraction of bioactive components from Salvia miltiorrhiza by natural deep eutectic solvents. Molecules 25, 140. 10.3390/molecules25010140 31905777 PMC6983008

[B45] Huang Y. Yang J. Zhao Y. Yu L. He Y. Wan H. (2021). Screening, optimization, and bioavailability research of natural deep eutectic solvent extracts from radix pueraria. Molecules 26, 729. 10.3390/molecules26030729 33572490 PMC7866862

[B46] Jeliński T. Przybyłek M. Cysewski P. (2019). Natural deep eutectic solvents as agents for improving solubility, stability and delivery of curcumin. Pharm. Res. 36, 1–10. 10.1007/s11095-019-2643-2 PMC6546644 31161340

[B47] Ji L. Du Q. Li Y. T. Hu W. (2016). Puerarin inhibits the inflammatory response in atherosclerosis via modulation of the nf-κb pathway in a rabbit model. Pharmacol. Rep. 68, 1054–1059. 10.1016/j.pharep.2016.06.007 27505855

[B48] Jin S. Y. (1988). Science of processing Chinese materia medica. Jiangsu: Jiangsu Science and Technology Press.

[B49] Juneidi I. Hayyan M. Mohd Ali O. (2016). Toxicity profile of choline chloride-based deep eutectic solvents for fungi and *Cyprinus carpio* fish. Environ. Sci. Pollut. Res. 23, 7648–7659. 10.1007/s11356-015-6003-4 26743645

[B50] Kelley S. P. Narita A. Holbrey J. D. Green K. D. Reichert W. M. Rogers R. D. (2013). Understanding the effects of ionicity in salts, solvates, co-crystals, ionic co-crystals, and ionic liquids, rather than nomenclature, is critical to understanding their behavior. Cryst. Growth Des. 13, 965–975. 10.1021/cg4000439

[B51] Khadka P. Ro J. Kim H. Kim I. Kim J. T. Kim H. (2014). Pharmaceutical particle technologies: An approach to improve drug solubility, dissolution and bioavailability. Asian J. Pharm. Sci. 9, 304–316. 10.1016/j.ajps.2014.05.005

[B52] Kim J. B. Paik Y. S. (1997). Stability of carthamin from *Carthamus tinctorius* in aqueous solution: pH and temperature effects. Arch. Pharm. Res. 20, 643–646. 10.1007/bf02975225 18982273

[B53] Kim S. J. Kim M. C. Lee B. J. Park D. H. Hong S. H. Um J. Y. (2010). Anti-Inflammatory activity of chrysophanol through the suppression of NF-kappaB/caspase-1 activation *in vitro* and *in vivo* . Molecules 15, 6436–6451. 10.3390/molecules15096436 20877234 PMC6257778

[B54] Komaikul J. Mangmool S. Putalun W. Kitisripanya T. (2021). Preparation of readily-to-use stilbenoids extract from *Morus alba* callus using a natural deep eutectic solvent. Cosmetics 8, 91. 10.3390/cosmetics8030091

[B55] Kong W. Wei J. Abidi P. Lin M. Inaba S. Li C. (2004). Berberine is a novel cholesterol-lowering drug working through a unique mechanism distinct from statins. Nat. Med. 10, 1344–1351. 10.1038/nm1135 15531889

[B56] Kowalczyk E. Krzesiński P. Kura M. Niedworok J. Kowalski J. Błaszczyk J. (2006). Pharmacological effects of flavonoids from Scutellaria baicalensis. Przegląd Lek. 63, 95–96. 16967717

[B57] Kumar A. K. Sharma S. Shah E. Patel A. (2018). Technical assessment of natural deep eutectic solvent (NADES) mediated biorefinery process: A case study. J. Mol. Liq. 260, 313–322. 10.1016/j.molliq.2018.03.107

[B58] Lanjekar K. J. Rathod V. K. (2021). Application of ultrasound and natural deep eutectic solvent for the extraction of glycyrrhizic acid from *Glycyrrhiza glabra*: Optimization and kinetic evaluation. Ind. Eng. Chem. Res. 60, 9532–9538. 10.1021/acs.iecr.1c00862

[B59] Li C. Lin G. Zuo Z. (2011). Pharmacological effects and pharmacokinetics properties of *Radix Scutellariae* and its bioactive flavones. Biopharm. Drug Dispos. 32, 427–445. 10.1002/bdd.771 21928297

[B60] Li J. Ding L. Song B. Xiao X. Qi M. Yang Q. (2016). Emodin improves lipid and glucose metabolism in high fat diet-induced obese mice through regulating SREBP pathway. Eur. J. Pharmacol. 770, 99–109. 10.1016/j.ejphar.2015.11.045 26626587

[B61] Li Y. Hsieh Y. Pan Z. Zhang L. Yu W. Wang B. (2020). Extraction of alkaloids from coptidis rhizoma via betaine‐based deep eutectic solvents. ChemistrySelect 5, 4973–4978. 10.1002/slct.202000865

[B62] Liao Y. Liang F. Liu H. Zheng Y. Li P. Peng W. (2018). Safflower yellow extract inhibits thrombus formation in mouse brain arteriole and exerts protective effects against hemorheology disorders in a rat model of blood stasis syndrome. Biotechnol. Biotechnol. Equip. 32, 487–497. 10.1080/13102818.2018.1429310

[B63] Liu Y. Brent Friesen J. McAlpine J. B. Lankin D. C. Chen S. N. Pauli G. F. (2018). Natural deep eutectic solvents: Properties, applications, and perspectives. J. Nat. Prod. 81, 679–690. 10.1021/acs.jnatprod.7b00945 29513526 PMC5913660

[B64] Lu Q. Y. Ma J. Q. Duan Y. Y. Sun Y. Yu S. Li B. (2019). Carthamin yellow protects the heart against ischemia/reperfusion injury with reduced reactive oxygen species release and inflammatory response. J. Cardiovasc. Pharmacol. 74, 228–234. 10.1097/fjc.0000000000000710 31356540

[B65] Lu Y. Jiang J. G. (2013). Application of enzymatic method in the extraction and transformation of natural botanical active ingredients. Appl. Biochem. Biotechnol. 169, 923–940. 10.1007/s12010-012-0026-9 23292902

[B66] Ma C. M. Wei Y. Wang Z. G. Hattori M. (2009). Triterpenes from Cynomorium songaricium-analysis of HCV protease inhibitory activity, quantification, and content change under the influence of heating. J. Nat. Med. 63, 9–14. 10.1007/s11418-008-0267-7 18600299

[B67] Mano F. Martins M. Sá-Nogueira I. Borges J. P. (2017). Production of electrospun fast-dissolving drug delivery systems with therapeutic eutectic systems encapsulated in gelatin. AAPS PharmSciTech 18, 2579–2585. 10.1208/s12249-016-0703-z 28236268

[B68] Martins M. A. R. Crespo E. A. Pontes P. V. Silva L. P. Bulow M. Maximo G. J. (2018). Tunable hydrophobic eutectic solvents based on terpenes and monocarboxylic acids. ACS Sustain. Chem. Eng. 6, 8836–8846. 10.1021/acssuschemeng.8b01203

[B69] Mbous Y. P. Hayyan M. Wong W. F. Looi C. Y. Hashim M. A. (2017). Unraveling the cytotoxicity and metabolic pathways of binary natural deep eutectic solvent systems. Sci. Rep. 7, 41257. 10.1038/srep41257 28145498 PMC5286504

[B70] Oomen W. W. Begines P. Mustafa N. R. Wilson E. G. Verpoorte R. Choi Y. H. (2020). Natural deep eutectic solvent extraction of flavonoids of *Scutellaria baicalensis* as a replacement for conventional organic solvents. Molecules 25, 617. 10.3390/molecules25030617 32023899 PMC7038101

[B71] Ou-Yang S. H. Jiang T. Zhu L. Yi T. (2018). *Dioscorea nipponica* makino: A systematic review on its ethnobotany, phytochemical and pharmacological profiles. Chem. Cent. J. 12, 57. 10.1186/s13065-018-0423-4 29748731 PMC5945570

[B72] Pan Z. Bo Y. Liang Y. Lu B. Zhan J. Zhang J. (2021). Intermolecular interactions in natural deep eutectic solvents and their effects on the ultrasound-assisted extraction of artemisinin from Artemisia annua. Artemisia annua J. Mol. Liq. 326, 115283. 10.1016/j.molliq.2021.115283

[B73] Pandey N. Pandey-Rai S. (2016). Updates on artemisinin: An insight to mode of actions and strategies for enhanced global production. Protoplasma 253, 15–30. 10.1007/s00709-015-0805-6 25813833

[B74] Park C. W. Kim J. Y. Rhee Y. S. Oh T. O. Ha J. M. Choi N. Y. (2012). Preparation and valuation of a topical solution containing eutectic mixture of itraconazole and phenol. Arch. Pharm. Res. 35, 1935–1943. 10.1007/s12272-012-1110-y 23212635

[B75] Patel D. Chaudhary S. A. Parmar B. Bhura N. (2012). Transdermal drug delivery system: A review. Pharma Innovation 1. 66 Available at: www.thepharmajournal.com .

[B76] Qiao J. Ji D. Sun S. Zhang G. Liu X. Sun B. (2018). Oral bioavailability and lymphatic transport of pueraria flavone-loaded self-emulsifying drug-delivery systems containing sodium taurocholate in rats. Pharmaceutics 10, 147. 10.3390/pharmaceutics10030147 30189624 PMC6161070

[B77] Qu W. Hakkinen R. Allen J. D'Agostino C. Abbott A. P. (2019). Globular and fibrous proteins modified with deep eutectic solvents: Materials for drug delivery. Molecules 24, 3583. 10.3390/molecules24193583 31590314 PMC6804121

[B78] Rácz G. Olasz Á. Rozsnyik Z. Ferenczi G. (2012). A modern approach to traditional Chinese medicine. IFAC Proc. Vol. 45, 196–200. 10.3182/20120829-3-HU-2029.00049

[B79] Ribeiro B. D. Florindo C. Iff L. C. Coelho M. A. Z. Marrucho I. M. (2015). Menthol-based eutectic mixtures: Hydrophobic low viscosity solvents. ACS Sustain. Chem. Eng. 3, 2469–2477. 10.1021/acssuschemeng.5b00532

[B80] Roda A. Santos F. Matias A. A. Paiva A. Duarte A. R. C. (2020). Design and processing of drug delivery formulations of therapeutic deep eutectic systems for tuberculosis. J. Supercrit. Fluids 161, 104826. 10.1016/j.supflu.2020.104826

[B81] Romero A. Santos A. Tojo J. Rodriguez A. (2008). Toxicity and biodegradability of imidazolium ionic liquids. J. Hazard. Mat. 151, 268–273. 10.1016/j.jhazmat.2007.10.079 18063302

[B82] Shi W. Wang Y. Li J. Zhang H. Ding L. (2007). Investigation of ginsenosides in different parts and ages of *Panax ginseng* . Food Chem. 102, 664–668. 10.1016/j.foodchem.2006.05.053

[B83] Shikov A. N. Kosman V. M. Flissyuk E. V. Smekhova I. E. Elameen A. Pozharitskaya O. N. (2020). Natural deep eutectic solvents for the extraction of phenyletanes and phenylpropanoids of *Rhodiola rosea* L. Molecules 25, 1826. 10.3390/molecules25081826 32316279 PMC7221623

[B84] Smith E. L. Abbott A. P. Ryder K. S. (2014). Deep eutectic solvents (DESs) and their applications. Chem. Rev. 114, 11060–11082. 10.1021/cr300162p 25300631

[B114] Spinozzi S. Colliva C. Camborata C. Roberti M. Ianni C. Neri F. (2014). Berberine and its metabolites: relationship between physicochemical properties and plasma levels after administration to human subjects. J. Nat. Prod. 77 (4), 776–772. 10.1021/np400607k 24593257

[B85] Srikrishna D. Godugu C. Dubey P. K. (2018). A review on pharmacological properties of coumarins. Mini-Rev. Med. Chem. 18, 113–141. 10.2174/1389557516666160801094919 27488585

[B86] Sut S. Faggian M. Baldan V. Poloniato G. Castagliuolo I. Grabnar I. (2017). Natural deep eutectic solvents (NADES) to enhance berberine absorption: An *in vivo* pharmacokinetic study. Molecules 22, 1921. 10.3390/molecules22111921 29117131 PMC6150298

[B87] Tian M. Yan H. Row K. H. (2008). Extraction of glycyrrhizic acid and glabridin from licorice. Int. J. Mol. Sci. 9, 571–577. 10.3390/ijms9040571 19325770 PMC2635700

[B88] van Osch D. J. G. P. Dietz C. Van Spronsen J. Gallucci F. Martin V. S. A. Tuinier R. (2019). A search for natural hydrophobic deep eutectic solvents based on natural components. ACS Sustain. Chem. Eng. 7, 2933–2942. 10.1021/acssuschemeng.8b03520

[B89] Walden P. (1914). Ueber die Molekulargrösse und elektrische Leitfähigkeit einiger geschmolzenen salze. Bull. Acad. Imper Sci. 8, 405–422.

[B90] Wan J. B. Yang F. Q. Li S. P. Wang Y. T. Cui X. M. (2006). Chemical characteristics for different parts of *Panax notoginseng* using pressurized liquid extraction and HPLC-ELSD. J. Pharm. Biomed. Anal. 41, 1596–1601. 10.1016/j.jpba.2006.01.058 16522361

[B91] Wang H. Ma X. Cheng Q. Xi X. Zhang L. (2018). Deep eutectic solvent-based microwave-assisted extraction of baicalin from scutellaria baicalensis georgi. J. Chem. 5, 1–10. 10.1155/2018/9579872 PMC6321484 30544548

[B92] Wang W. Cai Y. Liu Y. Zhao Y. Feng J. Liu C. (2017). Microemulsions based on paeonol-menthol eutectic mixture for enhanced transdermal delivery: Formulation development and *in vitro* evaluation. Artif. Cells Nanomed. Biotechnol. 45, 1241–1246. 10.1080/21691401.2016.1226178 27600884

[B93] Wang Y. Hu Y. Wang H. Tong M. Gong Y. (2020a). Green and enhanced extraction of coumarins from *Cortex Fraxini* by ultrasound-assisted deep eutectic solvent extraction. J. Sep. Sci. 43, 3441–3448. 10.1002/jssc.202000334 32579249

[B94] Wang Y. Peng B. Zhao J. Wang M. Zhao L. (2020b). Efficient extraction and determination of prenylflavonol glycosides in *Epimedium pubescens* Maxim. using deep eutectic solvents. Phytochem. Anal. 31, 375–383. 10.1002/pca.2904 31773856

[B95] Wikene K. O. Bruzell E. Tønnesen H. H. (2015). Characterization and antimicrobial phototoxicity of curcumin dissolved in natural deep eutectic solvents. Eur. J. Pharm. Sci. 80, 26–32. 10.1016/j.ejps.2015.09.013 26410725

[B96] Wu Y. C. Wu P. Li Y. B. Liu T. C. Zhang L. Zhou Y. H. (2018). Natural deep eutectic solvents as new green solvents to extract anthraquinones from *Rheum palmatum* L. Rheum. Palmatum L. RSC Adv. 8, 15069–15077. 10.1039/c7ra13581e 35541349 PMC9079993

[B97] Xiao S. Wang L. Han W. Gu L. Cui X. Wang C. (2022). Novel deep eutectic solvent–hydrogel systems for synergistic transdermal delivery of Chinese herb medicine and local treatments for rheumatoid arthritis. Pharm. Res. 39, 2431–2446. 10.1007/s11095-022-03239-5 35359240

[B98] Xie Y. Liu H. Lin L. Zhao M. Wu Y. Zhang Y. (2019). Application of natural deep eutectic solvents to extract ferulic acid from *Ligusticum Chuanxiong* Hort with microwave assistance. RSC Adv. 9, 22677–22684. 10.1039/c9ra02665g 35519449 PMC9067139

[B99] Xuan J. Wu X. Qi J. Zhuang J. (2021). Application of natural deep eutectic solvents in pharmaceutics. Acta Pharm. Sin. 56, 146–157. 10.16438/j.0513-4870.2020-1148

[B100] Yang G. Y. Song J. N. Chang Y. Q. Wang L. Zheng Y. G. Zhang D. (2021). Natural deep eutectic solvents for the extraction of bioactive steroidal saponins from Dioscoreae Nipponicae Rhizoma. Molecules 26, 2079. 10.3390/molecules26072079 33916390 PMC8038615

[B101] Yang L. Li L. Hu H. Wan J. Li P. (2019a). Natural deep eutectic solvents for simultaneous extraction of multi-bioactive components from Jinqi Jiangtang preparations. Pharmaceutics 11, 18. 10.3390/pharmaceutics11010018 30621239 PMC6359283

[B102] Yang X. J. Zhang P. Lv W. Zhou T. Li P. Zhao M. (2019b). Aggregation behavior of imidazolium-based amino acid ionic liquid surfactants in aqueous solution: The effect of amino acid counterions. J. Surfactants Deterg. 22, 515–523. 10.1002/jsde.12270

[B103] Yong C. S. Jung S. H. Rhee J. D. Choi H. G. Lee B. J. Kim D. C. (2003). Improved solubility and *in vitro* dissolution of Ibuprofen from poloxamer gel using eutectic mixture with menthol. Drug Deliv. 10, 179–183. 10.1080/713840406 12944138

[B104] Yu J. B. Zhao Z. X. Peng R. Pan L. B. Fu J. Ma S. R. (2019). Gut microbiota-based pharmacokinetics and the antidepressant mechanism of paeoniflorin. Front. Pharmacol. 10, 268–279. 10.3389/fphar.2019.00268 30949054 PMC6435784

[B105] Zang Y. Y. Yang X. Chen Z. G. Wu T. (2020). One-pot preparation of quercetin using natural deep eutectic solvents. Process Biochem. 89, 193–198. 10.1016/j.procbio.2019.10.019

[B113] Zeng Y. J. Xu P. Yang H. R. Zong M. H. Lou W. Y. (2018). Purification of anthocyanins from saskatoon berries and their microencapsulation in deep eutectic solvents. LWT 95, 316–325. 10.1016/j.lwt.2018.04.087

[B106] Zhang J. Zhang J. Wang S. Yi T. (2018). Development of an oral compound pickering emulsion composed of nanocrystals of poorly soluble ingredient and volatile oils from traditional Chinese medicine. Pharmaceutics 10, 170. 10.3390/pharmaceutics10040170 30275390 PMC6321358

[B107] Zhang X. Zhou Y. Gu Y. E. (2019). Tanshinone IIA induces apoptosis of ovarian cancer cells *in vitro* and *in vivo* through attenuation of PI3K/AKT/JNK signaling pathways. Oncol. Lett. 17, 1896–1902. 10.3892/ol.2018.9744 30675253 PMC6341594

[B108] Zhao B. Y. Xu P. Yang F. X. Wu H. Zong M. H. Lou W. Y. (2015). Biocompatible deep eutectic solvents based on choline chloride: Characterization and application to the extraction of rutin from *Sophora japonica* . Sophora Jpn. ACS Sustain. Chem. Eng. 3, 2746–2755. 10.1021/acssuschemeng.5b00619

[B109] Zheng N. Shi Y. L. Ji Y. X. Lu W. (2014). Stability and transformation of astragaloside Ⅳ, Ⅲ and Ⅰ. Cent. South Pharm. 12, 1062. 10.7539/j.issn.1672-2981.2014.11.002

[B110] Zuo J. Geng S. Kong Y. Ma P. Fan Z. Zhang Y. (2021). Current progress in natural deep eutectic solvents for the extraction of active components from plants. Crit. Rev. Anal. Chem. 29, 1–22. 10.1080/10408347.2021.1946659 34324395

